# Peculiarities of Diagnostic Reliability—Nested PCR Versus SAT in the Identification of *Helicobacter pylori*

**DOI:** 10.3390/microorganisms13071498

**Published:** 2025-06-27

**Authors:** Barbora Šipková, Michaela Abrahamovská, Janka Klingová, Bianka Prokopová, Jana Krčmáriková, Iveta Cihová, Pavol Sulo

**Affiliations:** 1 Department of Biochemistry, Faculty of Natural Sciences, Comenius University, Ilkovicova 6, 842 15 Bratislava, Slovakia; barbora.sel@gmail.com (B.Š.); abrahamovska@ibiotech.sk (M.A.); janka.klingova@gmail.com (J.K.); prokopova32@uniba.sk (B.P.); 2 Synlab Slovakia s. r. o., Limbova 5, 831 01 Bratislava, Slovakia; jana.krcmarikova@synlab.sk; 3 Department of Track and Field, Faculty of Physical Education and Sports, Comenius University, Nábr. arm. gen. L. Svobodu 9, 814 69 Bratislava, Slovakia; iveta.cihova@uniba.sk

**Keywords:** *H. pylori*, stool antigen test, diagnostic reliability, nested PCR

## Abstract

*H. pylori* detection via the stool antigen test (SAT) requires 100 times more cells than nested PCR (NPCR) for a 454 bp amplicon, but is significantly more sensitive in identifying positive stool samples. To understand this contradiction, we developed an NPCR assay to amplify a shorter 148 bp segment of the 16S rRNA gene. The assay was extremely sensitive and reliable when adhering to particular rules commonly used in forensic laboratories. The SAT and NPCR for long and short amplicons were compared using stool samples from 208 gastroenterological patients, of which 27.9% were identified as positive according to the SAT and only 6.25% according to the 454 bp NPCR amplicon, but 51.0% in the short 148 bp NPCR. Among 100 asymptomatic volunteers, the prevalence was 35% in the SAT assay and 22% in the long NPCR, but as much as 66.6% of positives were determined in the short 148 bp NPCR. The specificity of the PCR product was determined via DNA sequencing, which confirmed *H. pylori*’s origin in all NPCR-positive samples. Apparently, the stool contains mostly short fragments of *H. pylori* DNA, and the most plausible explanation for the SAT/NPCR paradox is the degradation of *H. pylori* DNA in the digestive system.

## 1. Introduction

*H. pylori*, infecting nearly half of the global population, is a significant cause of chronic gastrointestinal tract (GIT) disease. Fecal–oral and oral–oral transmission routes are considered to be the most common. Due to gastroesophageal reflux, it is also transmitted between relatives, often in childhood. The infection persists and remains with the host for the remainder of its life if untreated [1,2,3,4]. In most cases, the infection causes mild gastritis, which remains mostly asymptomatic. In approximately 10% of infected individuals, stomach and duodenal ulcers develop. The chronic state increases the risk of developing duodenal and gastric cancer. Therefore, this bacterium was classified as a type “group 1 (definite carcinogen)” by the International Agency for Research on Cancer in 1994, alongside asbestos and benzopyrene [2,4,5,6]. Stomach cancer is the fifth most common malignancy in the world (812,000 cases in 2018) and is the third-leading cause of cancer death [2,5,7,8,9]. This high mortality rate is associated with early metastatic expansion through the lymphatic system. The eradication of *H. pylori* has significantly reduced the incidence of ulcers to almost zero, and a corresponding decrease in stomach cancer incidence has been observed in most European countries [2,5,7,8,9,10]. The infection has also been associated with several extragastric diseases, including idiopathic thrombocytopenic purpura, iron deficiency anemia, vitamin B12 deficiency, insulin resistance, and metabolic syndrome [2,5,10,11].

Regarding *H. pylori*-driven diseases, early diagnostics play a crucial role [2,5,12]. There are several clinical tests for *H. pylori* identification used differentially, depending on the method of medical examination used and considering country-specific preferences. The Maastricht V/Florence Consensus Report primarily recommends the ^13^C-urea breath test (UBT) and the stool antigen test (SAT) for consistent identification [2,5,12]. In patients with dyspepsia older than 50, upper GIT endoscopy is recommended [5]. UBT remains a crucial tool for *H. pylori* diagnosis both before and after eradication therapy. A monoclonal SAT, if properly validated, is a suitable test for use before and after treatment. However, all tests have specific limitations in certain patient groups [11,12]. The ^13^C-UBT detects *H. pylori* indirectly by measuring the activity of bacterial urease in the stomach. Sensitivity and specificity are reported to be over 95%. False-negative test results can occur due to different delta over baseline (DOB)values, bleeding, or if the patient has received a proton pump inhibitor (PPI) or antibiotics before the examination [8,10,11,12,13,14,15]. In an inexpensive immunochromatographic stool antigen test, *H. pylori* antigens are recognized by monoclonal antibodies, yielding a sensitivity of 68.7% and a specificity of 97.6%. However, these outcomes vary across different studies, primarily due to the methods used as the gold standard for comparison. The accuracy can be affected similarly to in the UBT if the patient has taken PPIs, and false-negative results may occur when the *H. pylori* count is low [8,10,11,12,13,14,15].

Nevertheless, the Cochrane review of hospital-based studies has shown that at least 3% of cases examined via UBT and 9% of patients tested via *H. pylori*-specific serology tests (SAT) should be considered false negatives [8,12,16].

If a patient’s medical condition requires endoscopy, *H. pylori* infection is examined in gastric biopsies by a rapid urease test (RUT), histology, or cultivation. A RUT depends on the ability to secrete the enzyme urease. The sensitivity of the RUT is considered to be 94% for histology and 80–95% for cultivation, with an approximate range of 70–80%. However, in our hands, it is possible to cultivate *H. pylori* from only about 8% of positive samples [12,15,16].

Molecular methods, particularly real-time PCR, whole genome sequencing, and digital PCR, are accepted mainly for detecting mutations associated with resistance to specific antibiotics, especially when gastric biopsies are reused [2,5,12]. However, approaches involving DNA amplification have not been widely accepted in medical practice due to their higher cost compared to the SAT and the associated technical demands. The other objections concern doubts about accuracy, as alterations in the primer binding site may produce false negatives; on the other hand, non-specific primers may generate false positives [12,15,17,18]. Additionally, some positive results in PCR assays were reported without confirmation via different methods [12,15,18]. There are also considerable doubts about interpreting PCR results from a single gene, although their reliability can be unambiguously verified via sequencing [12,15,18]. Nevertheless, DNA amplification is the method of choice for identifying different bacterial species, as several target genes are available for detection and identification. Target genes include those encoding urease (ureA and ureC/glmM), as well as fliI, hpaA, and hsp60. However, the target of choice is the gene encoding 16S rRNA, which is common to all bacterial species and contains species-specific variable regions [12,15,18].

Several PCR modifications, such as real-time PCR and droplet digital PCR (ddPCR), enable the rapid detection and quantification of target DNA [12,15,18,19]. However, the disadvantages of real-time PCR include high equipment costs, a high level of technical skill required, and an increased chance of false-negative results due to operator error resulting from improper assay development and data analysis [12,15,18].

The alternative of choice is nested PCR (NPCR), which includes two rounds of PCR reactions. The first reaction amplifies a larger DNA region that serves as a template in the second reaction, which then amplifies a narrower subregion [12,15,18]. Due to the use of two sets of primers, NPCR is more specific, allowing DNA to be amplified from samples with a smaller number of target molecules than in simple PCR [15,18]. Notably, most published NPCR systems lack the specificity and sensitivity required to detect low-density infections in complex specimens [18]. Despite this fact, we elaborated a nested PCR for a 454 bp amplicon and to unambiguously identify *H. pylori* in biopsy, stool, and saliva using primers targeted to variable regions of the 16S ribosomal RNA (rRNA) gene. The sensitivity for DNA from stomach biopsies was on the same scale as ^13^C-UBT, but it was three times lower for DNA from stool. In addition, NPCR sensitivity in stool was two times lower than in the SAT, although *H. pylori* detection via the SAT requires 100 times more cells than NPCR for a 454 bp amplicon [18]. The most plausible explanation is the degradation of *H. pylori* DNA in the digestive system, present in the stool [18]. Therefore, the purpose of this article was to develop a sensitive and specific NPCR assay for amplifying a shorter 100–150 bp segment of 16S rRNA and to compare its sensitivity with that of the standard SAT assay.

## 2. Materials and Methods

### 2.1. Patients and Samples

A total of 208 stool samples (77 men and 131 women; mean age, 47 years; range, 4–90 years) were collected by the SYNLAB laboratory in Bratislava and provided along with the results of the stool antigen tests (SATs). All these samples were collected from patients with gastroenterological problems in 2019. To study the occurrence of *H. pylori* in the asymptomatic population, we used stool samples from 44 volunteers from the Faculty of Natural Sciences, (21 men and 35 women; mean age: 37 years; range 3 to 72 years) and 56 volunteers from the Faculty of Physical Education and Sports, (37 men and 9 women; mean age: 33 years; range 18 to 76 years), Comenius university. All samples were stored in a freezer at −20 °C. Patients and volunteers were instructed not to take proton pump inhibitors or antibiotics for at least 2 months prior to sample collection. All procedures were conducted under the Declaration of Helsinki. The study was approved by the Ethics Committee of the Faculty of Natural Sciences, Comenius University. The participants provided written informed consent in accordance with the principles outlined in the Declaration of Helsinki.

A culture of *H. pylori* J493, with a partial 16S rRNA sequence identical to that of clinical isolate B12823 used in optimizing and spiking experiments, was provided by Dr. Silvia Vašková, Laboratories, Piešťany, s.r.o. Slovakia.

### 2.2. SAT

The presence of *H. pylori* in stool was determined using the “DIAQUICK” immunochromatographic test, which is based on monoclonal antibodies (DIALAB g.m.b.h. Wr. Neudorf, Austria), according to the manufacturer’s instructions. Some samples were also examined using the “SureScreen” test (Derby, UK) and the “Vidia” test (Vestec, Czech Republic).

### 2.3. DNA Isolation

DNA from the stool samples (180–220 mg) was obtained using the QIAamp^®^ Fast DNA Stool Mini Kit (Qiagen, Valencia, CA, USA) according to the manufacturer’s instructions. In the final step, the DNA was desorbed into 40 μL of AE solution. The extracted DNA was stored at −20 °C for future analysis.

### 2.4. Nested PCR

**Figure 1:**
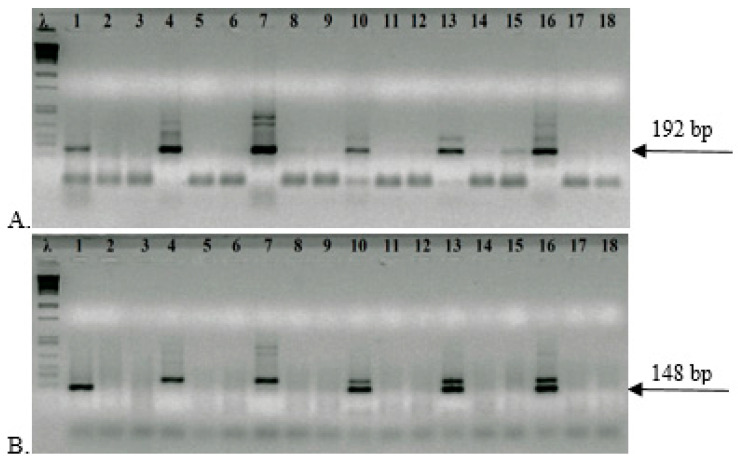
Representative data of the 148 bp amplicon nested PCR assay for *H. pylori* detection in stool samples. Lanes: λ λ/*Pst*I size marker; 1, 4, 7, 10, and 13 samples; 16 positive control; 2, 3, 5, 6, 8, 9, 11, 12, 14, 15, 17, and 18 negative controls (no DNA). (**A**) Amplified by external primers HeliS-trim/Satout (192 bp). (**B**) PCR product amplified by internal primers Hpup/Satin (148 bp), 2% agarose gel electrophoresis.

The primers for outer 16S rRNA 192 bp amplification were HeliS-trim 5′-AAGAACCTTACCTAGGCTTGACATTG-3′ and Satout 5′-AGTATCCTTAGAGTTCTCAGCATGACCT-3′. The primers for the second internal 148 bp amplification were Hpup 5′-TGAGAGAATCCGCTAGAAATAGTG-3′ and Satin 5′-CATGACCTGTTAGCAACTAAGAAAGG-3′. DNA was amplified using FIREPol^®^ DNA Polymerase (Solis BioDyne Tartu, Estonia) in a 25 μL solution that contained 1× buffer B; 0.2 mM of deoxynucleotides (dNTPs); 2.5 mM of MgCl_2_; 1X Super PCR Enhancer (S-Solution) (Solis BioDyne, Tartu, Estonia); 1 pmol/μL of each primer (HeliS-trim/Satout); 0.5 μL of DNA; and 0.2 U/μL of enzyme. For inconclusive cases, the DNA volume was increased up to 5 μL. The second PCR reaction included the same content, but with primers Hpup/Satin, and 1 μL of DNA from the first reaction was added. Amplification was performed in a thermal cycler (Eppendorf Mastercycler 5330, Eppendorf-Nethel-Hinz GmbH, Hamburg, Germany), at 94 °C for 3 min, 35 cycles of the external amplification reaction (94 °C, 45 s; 55 °C, 1 min; 72 °C, 1 min), 72 °C for 5 min, and a hold at 14 °C. The temperature cycle for the second reaction was the same, but the number of cycles was reduced to 25. To avoid any problem with contamination, two negative controls were introduced after every sample in triplicate experiments (sample–control–control; Figure 1). The NPCR for a 454 bp amplicon was performed as described in [18].

### 2.5. Analysis of PCR Products and Their DNA Sequences

The size of the PCR products was determined via electrophoresis in a 2% agarose gel and 1× Tris-borate-ethylenediaminetetraacetic acid (TBE) solution. λ/*Pst*I was used as a DNA size marker and to estimate the concentration of DNA in PCR products. All PCR products were sequenced after EPPiC Fast (A&A Biotechnology, Gdansk, Poland) treatment using the ABI–100 Avant and BigDye Terminator v3.1 Cycle Sequencing Kit (Applied Biosystems, Waltham, MA, USA). Sequences were edited in CHROMAS, trimmed to the same size, and compared with each other or with the sequences in the GenBank database using the blastn program at the National Center for Biotechnology Information (NCBI) (https://blast.ncbi.nlm.nih.gov/Blast.cgi?PROGRAM=blastn&BLAST_SPEC=GeoBlast&PAGE_TYPE=BlastSearch, accessed on 23 June 2025). Sequences were aligned using the Clustal W program [20], which is part of the CLC Genomics Workbench 9.5 package.

### 2.6. Other Procedures

Other procedures were carried out as described in [18].

## 3. Results

### 3.1. Presence of H. pylori Antigen in Stool

To understand why the SAT is 100 times less sensitive than NPCR for a 454 bp amplicon, we studied 208 stool samples from patients examined by a gastroenterologist, provided by the Synlab company, along with their results from the “Vidia” SAT (Vestec, Czech Republic). Initially, we retested the samples using the SAT from a different company, “DIAQUICK” (DIALAB g.m.b.h., Wr. Neudorf, Austria). Surprisingly, only 91.8% of the examined samples gave consistent data in both SATs; 34/208 were determined as positive and 157/208 as negative. Moreover, 8.2% (17/208) of the samples were positive in “DIAQUICK”, but not in “Vidia”. These data indicate that many patients in practice are misdiagnosed.

### 3.2. Presence of H. pylori Long 454 bp Amplicon in Stool

In previous work, Šeligová et al. (2020) [18], we elaborated and validated an NPCR test for the unambiguous identification of *H. pylori*, amplifying a 454 bp fragment of the 16S rRNA. However, only from half of the stool samples that were positive in the SAT were we able to amplify a longer segment of *H. pylori* DNA. The most likely explanation was that *H. pylori* DNA is degraded in stool to fragments smaller than 200 bp, just like food DNA, and longer fragments cannot be reliably amplified [21]. Therefore, we isolated DNA from stool and amplified a DNA fragment with a length of 454 bp using the so-called “long” NPCR. Using this NPCR, we attempted to amplify *H. pylori* DNA from all 208 samples. However, we were able to amplify *H. pylori*-specific DNA in only 13 samples, which represents a lower percentage of 6.25% compared to the previous paper [18].

### 3.3. Presence of H. pylori Short 148 bp Amplicon in Stool

To identify a shorter DNA fragment, we elaborated a new NPCR assay in which the external primers HeliS-trim /Satout amplified a 192 bp segment of the 16S rRNA gene, and internal primers Hpup/Satin DNA, which was 148 bp long. The primers were designed in 16S rRNA variable regions, and the conditions of amplification reactions were optimized as described previously in Šeligová et al. (2020) [18]. We also determined the detection limit by spiking 10-fold dilutions of *H. pylori* culture into negative stool samples. This was 100 cells/g stool, or 1.25 cells per reaction. To increase sensitivity, 5 μL of DNA template was added to the amplification reaction. In this case, to suppress the inhibition of the reaction, a 5-fold amount of DNA polymerase was added. Since the 10-fold increase in the amount of DNA and 5-fold amount of DNA polymerase increased the sensitivity of the reaction, we amplified two vials with negative controls after each sample containing the DNA template, which included all the components of the reaction mixture, but instead of DNA, only the same volume of water, and thus, we prevented the possibility of false-positive results (Figure 1).

If the signal also appeared in the negative control, we evaluated this sample as questionable and repeated the testing. Samples from which *H. pylori*-specific DNA could be repeatedly amplified, and the origin of the PCR product was confirmed via sequencing, were considered positive. Samples from which *H. pylori*-specific DNA could not be repeatedly amplified, but DNA was amplified by universal primers 341F/508R22, used for the simultaneous detection of eubacteria and archaea, were considered negative [22]. Moreover, 148 bp *H. pylori*-specific DNA was amplified in eight times more samples (106 vs. 13), indicating that stool contains mainly degraded *H. pylori* DNA (Table 1).

### 3.4. SAT Versus NPCR for Short 148 bp Amplicon

Table 1 summarizes the results of the comparison of the SAT and NPCR. Out of 208 gastroenterological patients, we identified only 58 positive cases using the SAT, yielding a prevalence of 27.9%. In contrast, the 106 positive cases identified via NPCR resulted in a prevalence of 51.0%.

If the short amplicon NPCR is considered a gold standard, the sensitivity of the SAT is only 54.7%, but the specificity is 93.6%. (Table 2).

### 3.5. SAT Versus NPCR for the Short 148 bp Amplicon in an Asymptomatic Population

To study the occurrence of *H. pylori* in the asymptomatic population, we examined stool samples from 44 volunteers from the Faculty of Natural Sciences (14 men and 30 women; mean age: 37 years; range 3 to 72 years) and 46 volunteers from the Faculty of Physical Education and Sports, Comenius University (37 men and 9 women; mean age: 33 years; range 18 to 76 years). The presence of *H. pylori* antigen was determined via the SAT “DIAQUICK” (DIALAB g.m.b.h. Wr. Neudorf, Austria). NPCR examined the presence of *H. pylori* DNA for a 148 bp amplicon, and the results are summarized in Table 3. According to the SAT, 35% of the population is colonized by *H. pylori*, while nested PCR indicates a colonization rate of 66%. Surprisingly, the prevalence is even higher than in the patients (Table 1), but it fits well with the estimated worldwide prevalence [2,4,7,23].

### 3.6. Variability of DNA Sequence from NPCR Short 148 bp Amplicons

**Figure 2:**
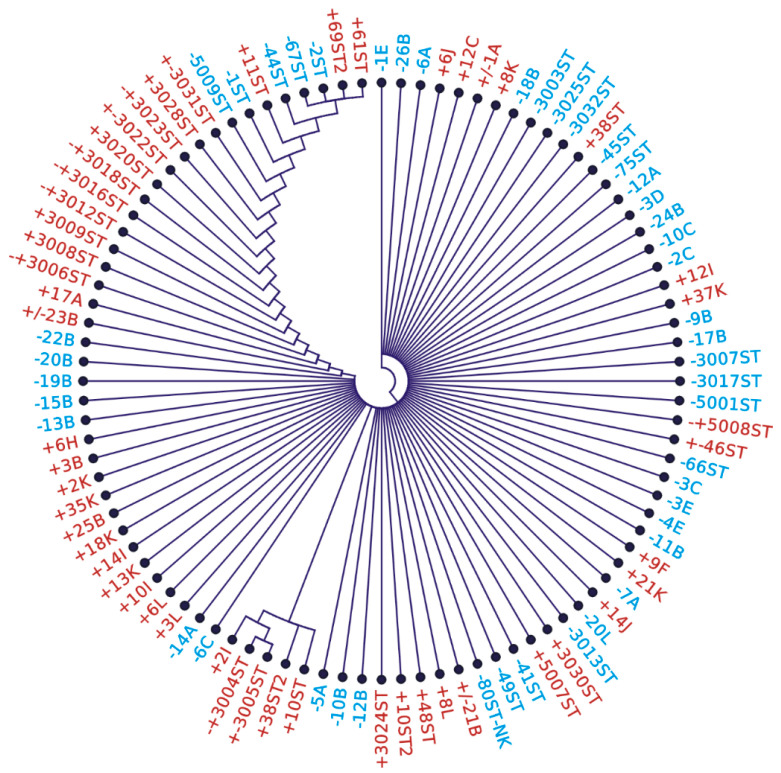
Circular cladogram showing the relatedness of *H. pylori* strains identified in stool samples. The cladogram was calculated from the comparison of the 94 bp 16S rDNA fragment (neighbor-joining algorithm) of 96 stool samples. Red highlights SAT-positive samples, blue highlights SAT-negative samples.

A total of 96 sequences amplified from stool (47 from an asymptomatic population and 49 from gastroenterology patients) were compared via Clustal X, but due to the short 94 nt DNA sequence, the comparison provided only seven polymorphic sites, generating 17 different variants (Figure 2; Supplementary Material Figure S1). The sequences could be divided into two main clades, which are roughly depicted by the left and right sides of the cladogram. Although the right clade contains more sequences from SAT-negatives, it cannot be concluded that SAT-negative samples contain *H. pylori* with a different sequence.

## 4. Discussion

The reason why *H. pylori* DNA cannot be consistently detected in stool via NPCR is related to its lower (threshold) occurrence and inability to survive independently of the stomach [18]. The most likely explanation concerning the detection in stool is the degradation of *H. pylori* cells and their DNA in the digestive tract. Food DNA tends to be degraded in stool samples into fragments smaller than 200 bp [21], whereas the size of the amplified region of the primary NPCR was 454 bp. A large part of *H. pylori* DNA is also degraded in stool; therefore, we sparsely amplified a 454 bp segment of the genome. To shed light on the degradation problem, we designed a new “short” NPCR assay that amplifies a shorter 148 bp section of the 16S rRNA gene. When we examined stool DNA from more than 300 individuals, short NPCR revealed five times more positive samples than the original long NPCR for a 454 bp amplicon. In addition, this assay was twice as sensitive as the SAT. The reliability of the 148 bp NPCR assay underscores the importance of sequencing the PCR product for the accurate interpretation of the results. The comparison with GenBank entries revealed exclusively *H. pylori* DNA, while the stool contained thousands of different bacterial species. These data demonstrate the tremendous specificity of NPCR [24].

To verify the reliability of the SAT, we compared the “Diaquick” and “Vidia” tests. It was astonishing when we found out that the results of SATs from different manufacturers differ significantly. It is worth noting that some samples, initially evaluated as negative, were found to be positive in subsequent tests, and vice versa. A good explanation is the threshold concentration and uneven distribution in the stool mentioned in Skrebinska et al. (2018) [25]. Alternatively, false-positive results may occur due to cross-reactivity and non-specific responses to antigens other than *H. pylori* [25,26]. Interestingly, we were unable to amplify seven samples that were positive via the SAT using the 148 bp NPCR, even in repeated experiments. We initially explained this by the interaction of the antibody present in the SAT with an antigen similar to that of *H. pylori*, which originates from another bacterium [25]. However, the most plausible explanation is DNA sequence divergence in primer annealing sites, and the nature of this inconsistency will be described in a following article (in preparation).

We attempted to identify an alternative pathogen by studying the differences in the microbiota of SAT-positive and SAT-negative patients using metagenomic analysis. Briefly, DNA isolated from stool samples was amplified using universal primers targeting the 16S rRNA gene, and the resulting libraries were sequenced using the Illumina MiSeq platform, as described by Gantuya et al. (2019) [22,27]. We did not find significant differences between negative-SAT and positive-NPCR samples, nor between negative-SAT and NPCR, or between positive-SAT and NPCR. Overall, it was a typical intestinal microbiota [22]. The most abundant were Bacteria; Bacteroidetes; Bacteroidia; Bacteroidales; Bacteroidaceae; and Bacteroides (0.9–37%). Bacteria; Firmicutes; Clostridia; Clostridiales; Ruminococcaceae; and Faecalibacterium (0–17.8%). Bacteria; Firmicutes; Clostridia; Clostridiales; Lachnospiraceae; and Agathobacter (0–24.8%). Bacteria; Firmicutes; Clostridia; Clostridiales; Ruminococcaceae; and Ruminococcus (0–17.9%) [27]. A significantly different microbiota was found in one sample with a negative-SAT and a positive-NPCR. In this case, the main groups present in the rest of the samples were absent, and their presence was compensated by the genus *Escherichia-Shigella*, which made up 35% of the total intestinal microbiota, and *Clostridium sensu stricto*, with 28.3%. *Shigella* and *Clostridium* are considered human pathogens that cause diarrhea, which could have an impact on the SAT assay. We also analyzed all raw sequence data for the presence of *H. pylori* 16S rRNA-specific DNA; however, we did not find any *H. pylori*-specific reads, which emphasizes the sensitivity of the 148 bp amplicon NPCR.

### Technical Merits

Testing for a short 148 bp amplicon is not a routine procedure. Initially, it was often observed that PCR products were present in nearly all negative controls, even when filter tips were used. The developed method was extremely sensitive, where opening the tubes and the spray effect was the source of amplification in the negative controls. To suppress the spray effect, we implemented measures typically used in forensic laboratories, where samples with extremely low DNA concentrations are amplified. To eliminate the spray effect, we used oil in the first PCR reaction. The individual operations were performed in three different rooms. The first was reserved for DNA isolation, the second for pipetting PCR reactions in a laminar flow box (Biohazard), and the third for electrophoresis and DNA analysis. Additionally, pipettes and centrifuges were explicitly designated for each operation and cleaned and sterilized every three months. Instead of polystyrene ice boxes, we used autoclavable plastic boxes. Two different centrifuges were used. One for mastermixes and the first PCR reaction, and another one exclusively for the second PCR reaction. With this regimen, *H. pylori*-specific DNA could be reliably identified.

During our work, we also encountered samples that exhibited signs of inhibition in the first amplification reaction. This phenomenon is common when DNA is isolated from stool. Stool samples contain many DNA inhibitors [28,29]. Although the kit used for isolation (QIAampR DNA Stool Mini Kit) contains components to eliminate inhibition, these components may not have been completely removed. We were able to recognize inhibited reactions based on the absence of the primer dimer signal (Figure 1A). The solution to this technical pitfall was a decrease of 10 times in the amount of DNA in the amplification reaction DNA (0.5 µL), re-isolating DNA from stool, or performing a second DNA elution from the column. In all cases, we were able to wholly or partially avoid inhibition in the retest. We are currently working on the one-vial modification of the 148 bp NPCR amplification, which we believe will become a routine procedure.

However, in practice, due to its price (EUR 2) and simplicity of handling, the method of choice is the SAT. The price of one nested PCR reaction can only be estimated because the cost of one amplification reaction in terms of chemicals and plastics is less than EUR 2. Six reactions are needed for reliable identification. If we consider the need for DNA isolation from stool, specialized equipment such as cyclers and laminar flow hoods, and the training of laboratory personnel, the cost will be significantly higher. A rough estimate is several tens of euros, and a significant initial investment is required. Therefore, we recommend testing the SAT first. In cases of persistent problems, SATs should be repeated, or other UBT methods should be used, while NPCR should be the last approach. However, several new emerging techniques based on completely new principles may displace it [30,31,32].

## 5. Conclusions

In conclusion, we elaborated a sensitive and specific NPCR assay to amplify a 148 bp segment of the 16S rRNA gene from stool samples. The assay is extremely sensitive and reliable when adhering to specific guidelines for a forensic laboratory, and is twice as sensitive as a routine SAT. In practice, we recommend the SAT for the initial screening. In case of negative results and persisting problems, repeated examination is advised with UBT and NPCR for the 148 bp amplicon.

## Figures and Tables

**Table 1:** Results of SAT versus NPCR in gastroenterology patients.

Patient Number	SAT	NPCR 148 bp Amplicon	NPCR 448 bp Amplicon
13	+	+	+
38	+	+	-
7	+	-	-
55	-	+	-
95	-	-	-
**208**	**58/208**	**106/208**	**13/208**

**Table 2:** SAT sensitivity and specificity related to NPCR (148 bp amplicon).

Sensitivity (%)	54.7
Specificity (%)	93.6
Positive predictive value	100.0
Negative predictive value	48.7

**Table 3:** Results of SAT versus NPCR in an asymptomatic population.

Number	SAT	NPCR 148 bp Amplicon	NPCR 448 bp Amplicon
22	+	+	+
13	+	+	-
31	-	+	-
34	-	-	-
**100**	**35/100**	**66/100**	**22/100**

## Data Availability

The original contributions presented in this study are included in the article/Supplementary Material. Further inquiries can be directed to the corresponding author.

## Supplementary Materials

The following supporting information can be downloaded at: https://www.mdpi.com/article/10.3390/microorganisms13071498/s1. Figure S1: Alignment of 17 *H. pylori* different sequences 94 nt long, obtained from 96 sequenced NPCR products.

## Abbreviations

The following abbreviations are used in this manuscript:

NPCR	Nested polymerase chain reaction
SAT	Stool antigen test
bp	Base pairs
UBT	Urea breath test
GIT	Gastrointestinal tract
PPI	Proton pump inhibitor
DOB	Delta over baseline
RUT	Rapid urease test
ddPCR	Droplet digital PCR
rRNA	Ribosomal RNA
