# Supplementary material for: Peculiarities of Diagnostic Reliability—Nested PCR Versus SAT in the Identification of Helicobacter pylori
Source: Microorganisms. 2025 Jun 27;13(7):1498. doi: 10.3390/microorganisms13071498 (PMC12301005; doi:10.3390/microorganisms13071498)
Supplement: Supplementary file 1 [file microorganisms-13-01498-s001.zip › microorganisms-3684045-supplementary.pdf]

Supplementary Figures

```
-6C         TGAGAGAATCCGCTAGAAATAGTGGAGTGTCTGGCTTGCCAGACCTTGAAAACAGGTGCT
+61ST       TGAGAGAATCCGCTAGAAATAGTGGAGTG-CTGGCTTGCCAGAGCTTGAAAACAGGTGST
+3008ST     TGAGAGAATCCGCTAGAAATAGTGGAGTGTCTGGCTTGCCAGACCTTGAAAACAGGTGCT
-+3012ST    TGAGAGAATCCGCTAGAAATAGTGGAGTGTCTGGCTTGCCAGACCTTGAAAACAGGTGCT
+3020ST     TGAGAGAATCCGCTAGAAATAGTGGAGTGTCTGGCTTGCCAGACCTTGAAAACAGGTGCT
-+3023ST    TGAGAGAATCCGCTAGAAATAGTGGAGTGTCTGGCTTGCCAGACCTTGAAAACAGGTGCT
-2ST        TGAGAGAATCCGCTAGAAATAGTGGAGTGTCTGGCTTGCCAGACCTTGAAAACAGGTGCT
+8L         TGAGAGAATCCGCTAGAAATAGTGGAGTGTCTGGCTTGCTAGACCTTGAAAACAGGTGCT
+10ST       TGAGAGAATCCGCTAGAAATAGTGGAGTGTCTGGCTTGCYAGACCTTGAAAACAGGTGCT
-3C         TGAGAGAATCCGCTAGAAATAGTGGAGTGTCTAGCTTGCTAGACCTTGAAAACAGGTGCT
-12A        TGAGAGAATCCGCTAGAAATAGTGGAGTGTCTAACTTGCTAGACCTTGAAAACAGGTGCT
-1E         TGAGAGAATCCGCTAGAAATAGTGGAGTGTCTAGCTTGCTAGACCTTGAAAACAGGTGCT
-11B        TGAGAGAATCCGCTAGAAATAGTGGAGTGTCTAGCTTGCTAGACCTTGAAAACAGGTGCT
-49ST       TGAGAGAATCCGCTAGAAATAGTGGAGTGTCTAGCTTGCTAGACCTTGAAAACAGGTGCT
+2I         TGAGAGAATCCGCTAGAAATAGTGGAGTGTCTRGCTTGCYAGACCTTGAAAACAGGTGCT
+38ST2      TGAGAGAATCCGCTAGAAATAGTGGAGTGTCTAGCTTGCYAGACCTTGAAAACAGGTGCT
-21B        TGAGAGAATCCGCTAGAAATAGTGGAGTGTCTAGCTTGCCAGACCTTGAAAACAGGTGCT
            ***************************** **  ***** *** ************** *

-6C         GCACGGCTGTCGTCAGCTCGTGTCGTGAGATGTT
+61ST       GCACGGCTGTCGTCAGCTCGTGTCGTGAGATGTT
+3008ST     GCACGGCTGTCGTCAGCTCGTGTCGTGAGATGTT
-+3012ST    GCACGGCTGTCGTCAGCTCGTGTCGTGAGATGTT
+3020ST     GCACGGCTGTCGTCAGCTCGTGTCGTGAGATGTT
-+3023ST    GCACGGCTGTCGTCAGCTCGTGTCGTGAGATGTT
-2ST        GCACGGCTGTCGTCAGCTCGTGTCGTGAGACGTT
+8L         GCACGGCTGTCGTCAGCTCGTGTCGTGAGATGTT
+10ST       GCACGGCTGTCGTCAGCTCGTGTCGTGAGATGTT
-3C         GCACGGCTGTCGTCAGCTCGTGTCGCGAGATGTT
-12A        GCACGGCTGTCGTCAGCTCGTGTCGTGAGATGTT
-1E         GCACGGCTGTCGTCAGCTCGTGTCGTGAGATGTT
-11B        GCACGGCTGTCGTCAGCTCGTGTCGTGAGATGTT
-49ST       GCACGGCTGTCGTCAGCTCGTGTCGTGAGATGTT
+2I         GCACGGCTGTCGTCAGCTCGTGTCGTGAGATGTT
+38ST2      GCACGGCTGTCGTCAGCTCGTGTCGTGAGATGTT
-21B        GCACGGCTGTCGTCAGCTCGTGTCGTGAGATGTT
            ************************* **** ***
```

Figure S1. Alignment of 17 *H. pylori* different sequences 94 nt long, obtained from 96 sequenced NPCR product. Variable nucleotides highlighted in red are in 7 polymorphic sites generating 17 different variants.
